# Validity and Reliability of the Spanish Version of the Children’s Self-Perceptions of Adequacy in and Predilection for Physical Activity (CSAPPA) Questionnaire for Primary School Children Aged 6 to 12 Years

**DOI:** 10.3390/children13070906

**Published:** 2026-07-08

**Authors:** Raquel Pastor-Cisneros, María Mendoza-Muñoz, José Francisco López-Gil, Jorge Carlos-Vivas

**Affiliations:** 1 Physical Activity for Education, Performance and Health (PAEPH) Research Group, Faculty of Sport Sciences, University of Extremadura, 10003 Cáceres, Spain; raquelpc@unex.es (R.P.-C.); jorgecv@unex.es (J.C.-V.); 2 Department of Communication and Education, Universidad Loyola Andalucía, 41704 Sevilla, Spain; mamendozam@unex.es; 3 School of Medicine, Universidad Espíritu Santo, Samborondón 0901952, Ecuador; 4 Faculty of Health Sciences, Universidad Autónoma de Chile, Temuco 4780000, Chile

**Keywords:** educational assessment, motivation, physical education, young children

## Abstract

Introduction: Understanding children’s physical self-concept is crucial, as it influences motivation, behavior, and physical activity (PA). The Spanish version of the Children’s Self-Perceptions of Adequacy in and Predilection for Physical Activity (CSAPPA) assesses the adequacy and predilection for PA, but it is not fully adapted for schoolchildren. Considering the developmental and contextual differences between children and adolescents, adapting and validating this instrument for younger populations is necessary. This study aimed to test the validity and reliability of the CSAPPA for schoolchildren aged 6–12 years (65 boys and 64 girls; mean age 9.63 ± 1.78 years). Method: Comprehension of the CSAPPA was examined through cognitive interviews, and reliability was assessed via a test–retest approach with 129 Spanish schoolchildren. Confirmatory factor analysis (CFA), Cronbach’s alpha, and the intraclass correlation coefficient (ICC) were used to evaluate validity and reliability. Results: CFA confirmed the three-factor structure (Adequacy, Predilection, Enjoyment) in children aged 6–12, with excellent fit indices: Chi-square divided by degrees of freedom (χ^2^/df) = 1.179; Root Mean Square Error of Approximation (RMSEA) = 0.037; Comparative Fit Index (CFI) = 0.961; Tucker–Lewis Index (TLI) = 0.953. Test–retest reliability ranged from questionable to good (Cronbach’s α = 0.622–0.871), except for items 8 and 14. The ICC values indicated moderate to near-perfect (ICC: 0.453–0.871) temporal stability; items 8 and 14 were excluded. Conclusions: The Spanish CSAPPA demonstrates validity and reliability for assessing self-perceptions related to PA motivation among schoolchildren aged 6–12. As part of the affective domain in the Spanish Physical Literacy Assessment for Children (SPLA-C) model, it shows promise for use in school and sport contexts, supporting the identification of needs and planning interventions to promote active participation and positive development. However, items 8 and 14 demonstrated poor psychometric performance and should be interpreted with caution, requiring further refinement before the instrument can be recommended without reservation.

## 1. Introduction

A person’s self-concept is their perception or belief about themselves, including their attributes, identity and general self-awareness. It encompasses the cognitive, emotional and social aspects of how people see themselves in relation to others and their environment [1]. According to Marsh and Shavelson [2], physical self-concept is “an individual’s perception and evaluation of their physical characteristics and motor skills”.

A previous study revealed that general physical self-concept, perceived competence, and perceived fitness can influence and be influenced by physical activity (PA) behavior among children and adolescents [3]. Given the existing decline in PA levels among children and adolescents [4], understanding the factors that influence these levels [5] is crucial for the development of effective programs that address these specific issues and increase PA.

According to Hay [6], two key self-perceptions are involved in the personal evaluation of one’s performance level: the perception of adequacy, which refers to one’s ability to achieve a goal, and predilection, which refers to a child’s intentionality in choosing a particular PA. To assess the adequacy and predilection for PA, the Children’s Self-Perceptions of Adequacy in and Predilection for Physical Activity (CSAPPA) [6,7,8,9,10] was designed, consisting of 20 items (7 adequacy, 10 predilection and 3 enjoyment items). This scale has been validated and translated into the Spanish population [11], reporting high adequacy and consistency among children and adolescents with an average age of 12 years.

Improving physical self-perception among schoolchildren requires context-specific assessment tools that consider their age, environment and developmental level [12]. These allow for the identification of real needs and the design of pedagogical strategies that promote positive body awareness and active participation in PA. There is a gap in the literature because no study has included an adaptation for this age group. In this sense, the original instrument warrants evaluation and adaptation before being applied to younger schoolchildren, as developmental differences in cognitive and linguistic abilities, together with contextual and sociocultural factors, may influence how questionnaire items are interpreted and answered [13,14]. Furthermore, younger children may experience difficulties understanding abstract concepts related to self-perceptions of competence and motivation, interpreting negatively worded statements, and processing more complex linguistic structures [14]. Consequently, instruments validated in older children and adolescents cannot automatically be assumed to function equivalently among younger schoolchildren. This will enable the appropriateness and predisposition to PA among schoolchildren to be assessed more accurately.

Given the significant differences in cognitive, linguistic and socioemotional development between school-aged children and adolescents [15], it is crucial to test whether the Spanish version of the CSAPPA is accessible and understandable for children in terms of language, structure and items. Therefore, this study aims to analyze the psychometric properties of the CSAPPA instrument for use with schoolchildren aged 6–12 years and to assess its validity and reliability in this population.

## 2. Materials and Methods

### 2.1. Study Design

This methodological, instrumental study was designed to examine the psychometric performance of the CSAPPA questionnaire in a younger population of primary school children. Although the instrument was originally developed for children and adolescents with a mean age of approximately 12 years, the present study sought to determine its validity and reliability when administered to children aged 6–12 years. The methodological procedures were based on established recommendations reported in previous research [16,17,18].

The research was carried out in two consecutive phases:

(1) Cognitive interviews to assess comprehensibility

The first phase focused on evaluating whether the questionnaire items were understandable and appropriate for the target age group. Cognitive interviews were conducted individually using a verbal probing approach to explore how children interpreted each item, understood its meaning, and selected their responses. Each interview lasted approximately 25 min.

The pilot sample consisted of 17 children enrolled in Years 2 to 6 of primary education (four from Year 2, four from Year 3, three from Year 4, three from Year 5, and three from Year 6). This sample size was established in accordance with World Health Organization recommendations and guidance from expert panels. Although 20 children were initially invited to participate, three did not complete the interview process. Interview notes were recorded by the researcher and examined descriptively to identify common difficulties related to comprehension, interpretation, or ambiguous wording. As this phase was exploratory in nature, the objective was to detect relevant comprehension issues rather than achieve theoretical saturation.

(2) Test–retest reliability assessment

To examine the temporal stability of the instrument, a subgroup of participants completed the questionnaire on two occasions separated by a 15-day interval. Test–retest reliability was subsequently evaluated by comparing the responses obtained at both time points.

Together, these two phases enabled the assessment of the questionnaire’s content validity, age-appropriate comprehensibility, and temporal stability in a sample of primary school children. The final version of the instrument is available in Supplementary Material S1.

### 2.2. Participants

A total of 129 primary school students (65 boys and 64 girls) between 6 and 12 years of age participated in the study. The sample was recruited from several public and private schools in Extremadura, Spain, encompassing children from a variety of socio-economic contexts. Participants were selected through convenience sampling. Eligibility criteria required that students: (a) were enrolled in primary education, (b) had written informed consent provided by a parent or legal guardian, and (c) demonstrated adequate reading comprehension to understand and complete the questionnaire independently.

### 2.3. Ethics Approval

The study protocol received ethical approval from the Bioethics and Biosafety Committee of the University of Extremadura (approval No. 288/2024). All research procedures complied with the ethical principles outlined in the latest revision of the Declaration of Helsinki, adopted at the 75th General Assembly of the World Medical Association (Helsinki, Finland, 2024), as well as with the provisions of Spanish Law 14/2007 on Biomedical Research. Written informed consent was obtained from the parents or legal guardians of all participants prior to their inclusion in the study.

### 2.4. Instrument

Data were collected using the Spanish version [11] of the original CSAPPA questionnaire [6], which is designed to assess three dimensions: appropriateness, predilection, and enjoyment. The scale consisted of 20 items (seven on appropriateness, ten on predilection, and three on enjoyment), asking respondents to indicate on a Likert-type scale which option most closely matched them, with options ranging from 1 (‘Not true for me’) to 4 (‘Completely true for me’)

Given that the Spanish version of the questionnaire is based on an established structure for 12-year-olds, a confirmatory factor analysis (CFA) was performed to determine whether this structure remains valid for a new population aged 6–12. A panel of experts reviewed the content of each item, and cognitive interviews were conducted with a pilot group of children (n = 17) to check the clarity, understanding and appropriateness of each item. Notably, no changes were made to the wording of the items following the cognitive interviews.

### 2.5. Statistical Analyses

All collected data were entered into a dedicated research database, and participants’ personal information was anonymized before analysis. Statistical analyses were carried out using IBM SPSS Statistics version 25.0 (IBM Corp., Armonk, NY, USA), while AMOS version 23.0.0 (IBM Corp., Armonk, NY, USA) was employed to perform the CFA.

The factorial structure of the Spanish version of the CSAPPA questionnaire was examined using Maximum Likelihood (ML) estimation. Although responses were obtained on a four-point Likert scale, ML estimation was selected because of its demonstrated robustness to moderate violations of normality and its extensive use in psychometric validation studies. Nevertheless, the ordinal nature of the response format and the relatively modest sample size should be considered when interpreting the findings, as estimators specifically developed for ordinal data; e.g., Weighted Least Squares Mean and Variance adjusted (WLSMV) may provide more precise parameter estimates.

The CFA included all questionnaire items as observed indicators. Model fit was evaluated using several complementary goodness-of-fit indices: (1) the chi-square statistic (with non-significant values indicating acceptable fit; *p* > 0.05) [19], (2) the root mean square error of approximation (RMSEA) [20], (3) the comparative fit index (CFI), (4) the Tucker–Lewis index (TLI), and (5) the chi-square per degree of freedom ratio (χ^2^/df) [21].

Temporal stability was examined using a test–retest design in which participants completed the questionnaire on two occasions separated by a 15-day interval. Prior to the reliability analyses, the Shapiro–Wilk and Levene tests were used to evaluate the assumptions of normality and homogeneity of variance, respectively. Descriptive statistics are presented as means and standard deviations (SD) for both assessment points.

Internal consistency was assessed using Cronbach’s alpha coefficient for both the individual dimensions and the overall questionnaire. Alpha coefficients were interpreted according to the classification proposed by Glen [22]; values below 0.50 were considered unacceptable, 0.50–0.60 poor, 0.60–0.70 questionable, 0.70–0.80 acceptable, 0.80–0.90 good, and values above 0.90 excellent.

Test–retest reliability was quantified using the intraclass correlation coefficient (ICC) with corresponding 95% confidence intervals [23]. Agreement between the two administrations was estimated using a two-way random-effects model based on single measurements and absolute agreement. ICC values were interpreted following the benchmarks established by Landis and Koch [24], whereby values below 0.20 indicate slight agreement, 0.21–0.40 fair agreement, 0.41–0.60 moderate agreement, 0.61–0.80 substantial agreement, and values above 0.80 almost perfect agreement.

To complement the relative reliability analyses, absolute reliability was evaluated by calculating the standard error of measurement (SEM) and the minimum detectable change (MDC) [25]. Finally, Spearman’s rank-order correlation coefficient was used to examine the relationship between each questionnaire item and the total CSAPPA score. Statistical significance was set at *p* ≤ 0.05 for all analyses.

## 3. Results

### 3.1. Confirmatory Factor Analysis (CFA)

**Figure 1:**
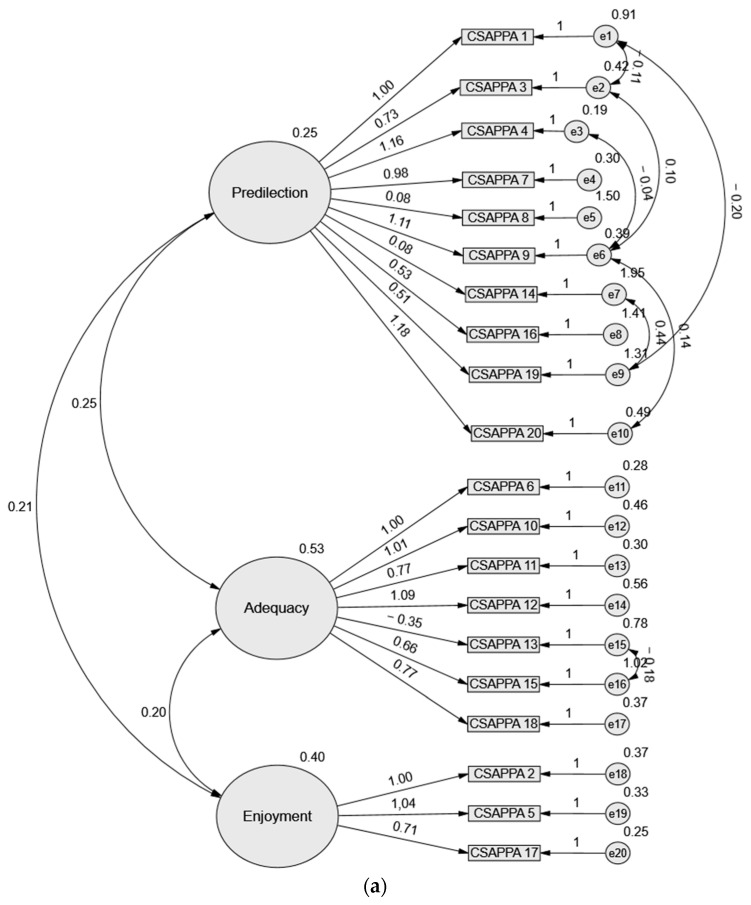

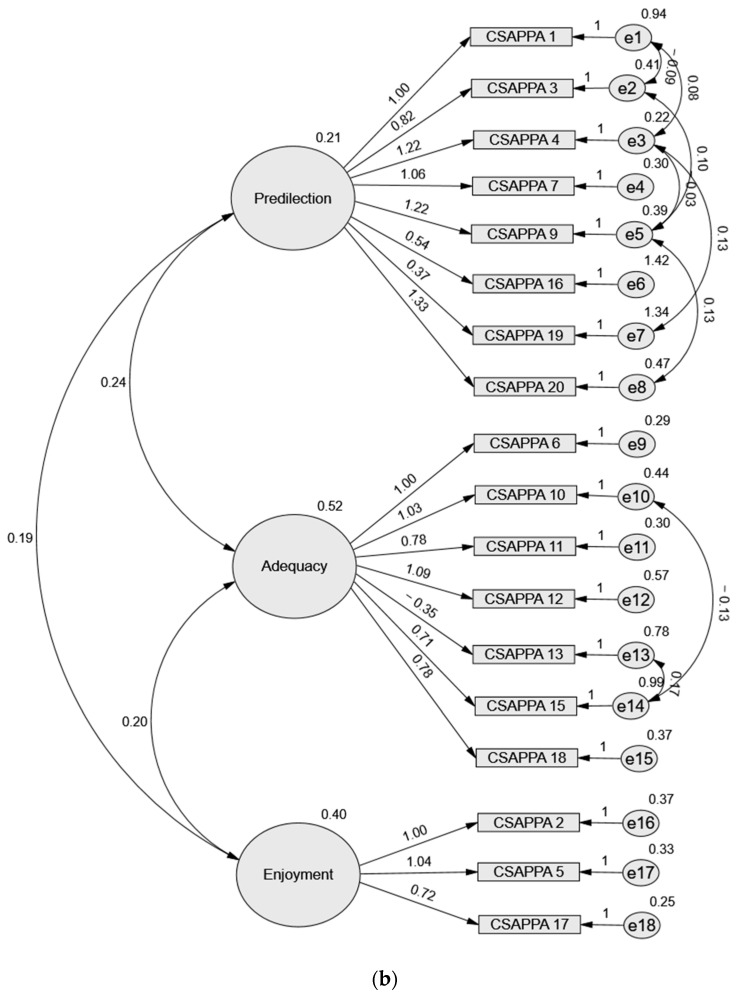
(**a**) CFA model for the original 20-item CSAPPA. (**b**) CFA model for the alternative 18-item CSAPPA excluding Items 8 and 14.

A confirmatory factor analysis was conducted with a total of 129 participants aged 9.63 (±1.78) years, of whom 50.4% were boys and 49.6% were girls. The resulting model for the original 20-item CSAPPA is presented in Figure 1a. Given the poor psychometric performance observed for Items 8 and 14 in the reliability analyses, an additional CFA was performed excluding these items. The resulting 18-item model is presented in Figure 1b.

A comparison of the goodness-of-fit indices obtained for both models is shown in Table 1. The original 20-item model (Figure 1a) demonstrated a good overall fit, with χ^2^/df = 1.179, and the chi-square test was non-significant (*p* = 0.061). Because no single fit index should be interpreted in isolation, model fit was evaluated using a combination of absolute, incremental, and parsimonious fit indices [26]. The comparative fit index (CFI) was 0.961 and the Tucker–Lewis’s index (TLI) was 0.953, both exceeding the recommended threshold of 0.95 for excellent fit [27]. Additionally, the root mean square error of approximation (RMSEA) was 0.037, well below the recommended cutoff value of 0.06, indicating minimal approximation error and a close fit between the proposed model and the observed data [28] and the standardized root mean square residual (SRMR) was 0.064, suggesting that the discrepancies between the observed covariance matrix and the one predicted by the model were small.

Given the poor psychometric performance of Items 8 and 14, an additional CFA was conducted excluding these two items (Figure 1b). The alternative 18-item model also demonstrated excellent fit, with χ^2^/df = 1.163, CFI = 0.972, TLI = 0.965, RMSEA = 0.036, and SRMR = 0.058.

As shown in Table 1, the exclusion of Items 8 and 14 resulted in only modest improvements across the fit indices. Although the alternative model yielded slightly higher CFI and TLI values and slightly lower RMSEA and SRMR values, the magnitude of these differences was small. Therefore, the complete 20-item version was retained for the primary analyses, while acknowledging the problematic psychometric performance of Items 8 and 14 and the need for their future refinement.

### 3.2. Test–Retest Reliability and Internal Consistency

The internal consistency, reproducibility and systematic differences in the CSAPPA are illustrated in Table 2. Overall, the internal consistency ranged from questionable to good for all the items and the total score of the questionnaire (Cronbach’s α ranged from 0.622 to 0.871), except for items 8 and 14, where it was unacceptable (Cronbach’s α < 0.5). All items at the initial and follow-up tests significantly correlated with total CSAPPA scores (r from 0.180 to 0.784), except for item 14 at the initial test (*p* > 0.05).

Reproducibility outcomes revealed moderate to near-perfect test–retest reliability for each item and the total CSAPPA score (ICC: 0.453–0.871), except for items 8 and 14, for which the agreement was slight (ICC < 0.2). The SEM and SEM% values for each item and the total CSAPPA score ranged from 0.34 to 3.29 and from 5.29 to 47.08, respectively. Similarly, the MDC and MDC% values for each item and the total CSAPPA score ranged from 0.94 to 9.13 and from 14.65 to 130.51, respectively.

Finally, comparison outcomes overall showed no significant differences for any of the items or the total CSAPPA score (*p* > 0.05), except for item 8 (*p* = 0.018) and item 14 (*p* < 0.001).

## 4. Discussion

This study examined the psychometric properties of the CSAPPA questionnaire (Spanish version) for use with schoolchildren aged 6 to 12 years. The results of the CFA provided evidence supporting the structural validity of the instrument in this population, with fit indices classified as excellent. In terms of reliability, the questionnaire demonstrated high internal consistency for the total score and acceptable to good values for most individual items. Furthermore, the test–retest analysis revealed acceptable to excellent temporal stability for most items and for the total score. However, items 8 and 14 exhibited unacceptable levels of internal consistency and test–retest reliability, along with significant differences across assessment times. Therefore, although the overall findings support the use of the CSAPPA for assessing perceived adequacy, predilection, and enjoyment toward physical activity among Spanish schoolchildren, these results should be interpreted with caution, as items 8 and 14 require further refinement before the instrument can be recommended without reservation.

The results of this study are highly consistent with those of previous research examining the psychometric properties of the CSAPPA questionnaire within different population contexts [29]. The model originally proposed by Hay [6] has been successfully replicated in the Spanish population [11], which suggests a theoretically sound and generalizable structure. In particular, the fit indices obtained in the present research not only confirm the adequacy of the model in Spanish schoolchildren aged 6 to 12 years but also exceed the levels of fit observed in adaptations of similar instruments [30,31]. Additionally, the CFA results of our study support the three-dimensional factor structure proposed by the original English version [6]. In our sample, the CFI and TLI values (CFI = 0.961, TLI = 0.953) were within the range considered “excellent” (≥0.95). These values also align with the RMSEA value (0.037), indicating a good fit of the model. An additional CFA excluding items 8 and 14 yielded only modest improvements in model fit indices, suggesting that although these items exhibited poor reliability, they did not substantially compromise the overall factorial structure of the instrument. Consequently, the original 20-item version was retained to preserve the conceptual coverage of the three-factor model, while recognizing the need for future refinement of these items. The overall pattern of fit indices supports the adequacy of the proposed model. Furthermore, the theoretical soundness and practical applicability of this questionnaire for assessing the three key dimensions (appropriateness, predilection and enjoyment), which are primarily based on perceived competence theory [32] and self-determination theory [33], are supported.

In terms of internal reliability, the Cronbach’s alpha values observed in this study are in the acceptable to excellent range (α = 0.622 to 0.871), which coincides with the values reported by the original questionnaire (α = 0.65 to 0.85) [6], as well as with the coefficients reported in previous validation studies of other motivation questionnaires [34,35]. However, as in previous studies that analyzed the psychometric properties of certain questionnaires [36,37], some items have demonstrated poor psychometric performance, particularly when they contain ambiguous or abstract formulations that are difficult for younger children to understand [38]. In this respect, the issues identified with items 8 and 14 align with the challenges previously documented in validation studies conducted in different populations. These studies emphasize the necessity of adjusting certain items to enhance comprehension and consistency among children [39].

The test–retest reliability of the CSAPPA yielded ICC values ranging from 0.453 to 0.871. These results suggest that the majority of items demonstrated temporal stability within the moderate to near-perfect range for both individual items and the total score. These findings are consistent with the results of previous CSAPPA validation studies [6,11] as well as with the validation of other questionnaires related to motivation and physical exercise in children [40,41], where ICCs fall within similar ranges or are lower than those obtained in this study. However, these findings should be interpreted in light of the poor performance observed for items 8 and 14, which showed limited temporal stability and substantial measurement error. The lower ICCs observed for certain items may be influenced by age-related variability in the cognitive and emotional development of younger children [42], who have not yet fully understood the concept of internal motivational states [43,44].

Despite the strong overall results, items 8 and 14 of the CSAPPA questionnaire demonstrated poor psychometric performance in terms of both internal consistency and temporal stability. Specifically, both items had Cronbach’s alpha values less than 0.50 and very low item–total correlations and intraclass correlation coefficients, indicating barely significant or even zero test–retest reliability. Furthermore, these were the only items that exhibited statistically significant differences between the initial and subsequent tests, suggesting the presence of systematic errors or comprehension difficulties in their formulation.

From a practical perspective, the elevated SEM and MDC values observed for these items indicate reduced measurement precision and a limited ability to detect meaningful changes over time. SEM reflects the amount of measurement error associated with an observed score, whereas MDC represents the minimum change required to be confident that a true change has occurred beyond measurement error. Consequently, the MDC percentages observed for items 8 (130.5%) and 14 (105.5%) suggest that substantial score changes would be necessary before a real change could be distinguished from measurement error, further questioning their suitability for use in this age group.

One possible explanation for this is that the content of these items does not fully align with the cognitive or linguistic development level of the participants, particularly given that the sample included children as young as six years old. Some terms or grammatical constructions may be too abstract or ambiguous for younger children [44,45], making them difficult to interpret and consequently affecting the reliability of the responses. Limited semantic understanding could therefore have led to inconsistent interpretations or random responses. Additionally, both items coincide with items that are scored inversely; i.e., a higher score is considered negative, and a lower score is considered positive, which may be detrimental to the results of our study [46]. Children may also experience difficulties processing abstract concepts and reverse-worded statements even when they appear to understand the literal meaning of the item during cognitive interviews. Therefore, verbal probing alone may not always be sufficiently sensitive to detect subtle response-processing difficulties associated with these types of items. This may explain why the cognitive interview phase did not identify the problems that later emerged during the psychometric analyses. Consequently, future adaptations should incorporate more extensive cognitive testing procedures, including larger pilot samples and multiple cognitive interviewing techniques, particularly when reverse-worded items are included. Given their consistently poor psychometric performance, items 8 and 14 should be prioritized for reformulation, replacement, or removal, and future validation studies should determine whether revised versions improve measurement performance while preserving the theoretical structure of the questionnaire.

### 4.1. Strength and Practical Implications

The findings of this study have relevant applied implications for sport science, physical education (PE), and public health. The validated Spanish version of the CSAPPA provides a psychometrically sound and developmentally appropriate instrument to assess perceived adequacy, predilection, and enjoyment toward PA in schoolchildren, key psychological determinants of sport participation and long-term adherence to active lifestyles. Given that childhood is a critical stage for establishing motivational patterns and movement behaviors, the availability of this tool allows researchers and practitioners to better understand the affective factors influencing engagement in both school-based PE and organized youth sports.

In practical terms, the CSAPPA can assist PE teachers and youth sport coaches in identifying children with low perceived competence, reduced enjoyment, or motivational vulnerability, which are factors commonly associated with disengagement and early sport dropout. Early identification may facilitate the implementation of targeted, inclusive, and developmentally appropriate interventions aimed at enhancing confidence, positive sport experiences, and sustained participation. From a public health perspective, systematically assessing these motivational constructs during primary school years can contribute to preventive strategies designed to increase physical activity levels and reduce sedentary behaviors and their associated health risks. Therefore, the Spanish CSAPPA represents not only a validated measurement instrument but also a practical resource to inform evidence-based sport- and school-based programs promoting active and healthy development in young people.

Notably, this study is consistent with a recent publication describing the development of the Spanish Physical Literacy Assessment for Children (SPLA-C), the first model for assessing physical literacy (PL) in Spain [47]. In this Delphi study, national experts agreed that the CSAPPA questionnaire should be included under the “motivation and confidence” component of the model. As part of the SPLA-C framework, the CSAPPA provides a valuable tool for schools and policymakers to track students’ motivation in the affective domain of PL. This insight can support the design of more comprehensive PE programs that go beyond motor skills to also foster students’ intrinsic motivation for PA.

Finally, practitioners should be aware that, although the original scoring procedure has been retained to preserve consistency with the original CSAPPA, the scores obtained for items 8 and 14 should be interpreted with caution because these items demonstrated consistently poor psychometric performance. Until further validation studies establish whether these items should be reformulated, replaced, or removed, decisions based on individual responses to these items should be avoided, and greater emphasis should be placed on the interpretation of the overall questionnaire scores and the remaining well-performing items.

### 4.2. Limitations and Future Line Research

Despite the positive results obtained regarding the validity and reliability of the Spanish version of the CSAPPA, several limitations should be acknowledged.

First, the sample was limited to schoolchildren from a single geographical region, which restricts the generalizability of the findings to other cultural and educational contexts within Spain. Ideally, future studies should include participants from different autonomous communities and a broader range of sociodemographic backgrounds. Moreover, socioeconomic status was not formally collected as part of the study protocol, limiting the characterization and representativeness of the sample. Future research should therefore incorporate socioeconomic indicators to better describe participant characteristics and improve the external validity of the findings.

A second limitation relates to the methodological aspects of the confirmatory factor analysis. Although the fit indices supported the proposed three-factor structure and recent structural equation modeling literature [26,48] suggests that sample size adequacy depends on model characteristics rather than fixed participant-to-item ratios, the relatively modest sample size may have affected the stability of some parameter estimates. Furthermore, no a priori sample size calculation or Monte Carlo simulation specifically designed for the proposed CFA model was conducted, which would have provided a stronger justification for sample adequacy. Therefore, the factorial structure should be considered preliminary until replicated in larger and independent samples.

In addition, the CFA was estimated using Maximum Likelihood despite the questionnaire employing a four-point Likert response scale. Although this approach is frequently used in psychometric validation studies, future research should replicate the analyses using estimation methods specifically designed for ordinal data, such as WLSMV. Likewise, multivariate normality was not formally assessed using Mardia’s coefficient or equivalent procedures, and future studies should evaluate this assumption and consider robust estimation methods where appropriate. Finally, although the present study addressed the main psychometric properties of the instrument, future validation studies would benefit from a more comprehensive evaluation following the complete COnsensus-based Standards for the selection of health Measurement Instruments (COSMIN) framework, including additional measurement properties and reporting standards.

Although the questionnaire was validated across the entire sample of children aged 6–12 years, this age range encompasses important cognitive, linguistic, and socioemotional developmental differences. Due to sample size constraints, measurement invariance analyses across age groups could not be performed. Future studies should therefore examine whether the factorial structure remains stable across different developmental stages and educational levels.

Second, the cross-sectional nature of the study prevents the assessment of long-term stability and sensitivity to change following educational interventions or physical activity programs. Longitudinal studies are needed to determine how children’s self-perceptions evolve over time and whether the instrument can detect meaningful changes resulting from intervention programs.

Finally, items 8 and 14 performed poorly across virtually all psychometric indicators analyzed. These findings are consistent with their low internal consistency, poor test–retest reliability, and high measurement error, indicating that these items should not be considered fully functional indicators in their current form. Although cognitive interviews suggested that participants generally understood the questionnaire content, the pilot phase involved a relatively small number of children, and the verbal probing approach may not have been sufficiently sensitive to detect subtle response-processing difficulties associated with reverse-worded items. The poor performance of these items may be related not only to their reverse-scored wording, but also to developmental, linguistic, and cognitive factors, as younger children may experience difficulties processing negation, abstract concepts, and reverse-worded statements even when they appear to understand the item during cognitive interviewing. Consequently, future studies should prioritize the reformulation, replacement, or removal of these items and incorporate more extensive cognitive testing procedures with larger pilot samples involving children from different age groups. Additionally, future validation studies should evaluate whether revised or alternative versions of the questionnaire, including versions excluding these items, improve the overall psychometric performance of the instrument while preserving its theoretical structure.

## 5. Conclusions

This study provides preliminary evidence supporting the structural validity and reliability of the Spanish version of the CSAPPA questionnaire in schoolchildren aged 6–12 years. The three-factor structure showed good overall fit, and most items demonstrated adequate internal consistency and temporal stability. However, items 8 and 14 consistently exhibited poor psychometric performance and should be interpreted with caution. These items require further refinement, replacement, or removal before the questionnaire can be recommended for routine use without reservation. Accordingly, future validation studies should prioritize the evaluation of revised versions of these items or alternative questionnaire structures to determine whether their psychometric performance can be improved while preserving the original theoretical framework. Despite these limitations, the Spanish CSAPPA represents a valuable instrument for assessing perceived adequacy, predilection, and enjoyment toward physical activity in schoolchildren and may support the identification of motivational needs and the planning of school- and sport-based interventions. Nevertheless, practitioners should interpret the results of items 8 and 14 with caution until further evidence regarding their performance becomes available.

Finally, the inclusion of the CSAPPA within the affective domain of the SPLA-C further supports its potential contribution to the comprehensive assessment of PL in Spain while highlighting the importance of continuing to refine the instrument in future validation studies.

## Figures and Tables

**Table 1:** CSAPPA goodness-of-fit indices comparative.

Indices	20-Items Version	18-Items Version (No Items 8 and 14)
χ^2^/df	1.179	1.163
p (χ^2^)	0.061	0.103
RMSEA	0.037	0.036
CFI	0.961	0.972
TLI	0.953	0.965
SRMR	0.064	0.058

χ^2^/df, minimum discrepancy per degree of freedom; P (χ^2^), chi-squared probability; RMSEA, root mean square error of approximation; CFI, comparative fit index; TLI, Tucker-Lewis index; SRMR, standardized root mean square residual.

**Table 2:** Reliability, test–retest, and systematic differences in CSAPPA.

Item	Test (n = 129)			Retest (n = 129)				Reliability					
	M	SD	Item-Total Correlation	M	SD	Item-Total Correlation	Cronbach’s α	ICC (95% CI)	*p* Value ^†^	SEM	SEM%	MDC	MDC%
Item 1	3.06	1.08	0.418 **	3.12	1.04	0.460 **	0.796	0.661 (0.552 to 0.748)	0.419	0.62	20.0	1.71	55.4
Item 2	3.42	0.88	0.437 **	3.41	0.86	0.544 **	0.761	0.616 (0.497 to 0.713)	0.908	0.54	15.8	1.49	43.8
Item 3	3.48	0.75	0.501 **	3.44	0.84	0.784 **	0.622	0.453 (0.304 to 0.580)	0.596	0.59	17.0	1.63	47.1
Item 4	3.60	0.73	0.614 **	3.67	0.69	0.576 **	0.871	0.771 (0.690 to 0.832)	0.144	0.34	9.3	0.94	25.9
Item 5	3.29	0.88	0.475 **	3.22	0.94	0.488 **	0.796	0.661 (0.552 to 0.748)	0.346	0.53	16.3	1.47	45.1
Item 6	3.33	0.90	0.637 **	3.31	0.93	0.681 **	0.808	0.680 (0.575 to 0.763)	0.904	0.52	15.6	1.43	43.2
Item 7	3.64	0.74	0.536 **	3.54	0.86	0.632 **	0.678	0.511 (0.372 to 0.627)	0.149	0.56	15.6	1.55	43.2
Item 8	2.64	1.23	0.180 *	2.29	1.19	0.209 *	0.153	0.080 (−0.087 to 0.245)	0.018	1.16	47.1	3.22	130.5
Item 9	3.43	0.85	0.604 **	3.38	0.88	0.699 **	0.634	0.466 (0.319 to 0.591)	0.552	0.63	18.6	1.75	51.5
Item 10	3.05	1.00	0.669 **	3.12	0.94	0.621 **	0.663	0.496 (0.354 to 0.616)	0.417	0.69	22.3	1.91	61.9
Item 11	3.40	0.79	0.565 **	3.43	0.84	0.621 **	0.659	0.493 (0.350 to 0.613)	0.666	0.58	17.0	1.61	47.1
Item 12	2.79	1.09	0.666 **	2.74	1.08	0.536 **	0.729	0.575 (0.446 to 0.680)	0.599	0.71	25.6	1.96	70.9
Item 13	1.67	0.92	−0.209 *	1.58	0.86	−0.315 **	0.692	0.529 (0.393 to 0.642)	0.264	0.61	37.6	1.69	104.2
Item 14	2.47	1.40	0.075	3.58	0.86	0.525 **	−0.115	−0.038 (−0.155 to 0.093)	<0.001	1.15	38.1	3.19	105.5
Item 15	3.06	1.12	0.394 **	3.00	1.13	0.537 **	0.689	0.527 (0.390 to 0.641)	0.519	0.77	25.5	2.14	70.8
Item 16	2.74	1.22	0.260 **	2.77	1.20	0.363 **	0.701	0.541 (0.407 to 0.653)	0.704	0.82	29.8	2.27	82.5
Item 17	3.61	0.68	0.203 **	3.57	0.80	0.447 **	0.858	0.752 (0.666 to 0.818)	0.398	0.37	10.3	1.02	28.5
Item 18	3.26	0.83	0.635 **	3.26	0.85	0.703 **	0.809	0.681 (0.577 to 0.764)	>0.999	0.47	14.6	1.32	40.3
Item 19	2.67	1.17	0.232 **	2.74	1.14	0.321 **	0.657	0.490 (0.347 to 0.611)	0.451	0.82	30.5	2.29	84.5
Item 20	3.39	0.92	0.602 **	3.37	0.94	0.605 **	0.781	0.643 (0.529 to 0.734)	0.823	0.56	16.4	1.54	45.6
Total CSAPPA score	62.00	8.56	N/A	62.59	9.78	N/A	0.871	0.871 (0.817 to 0.909)	0.282	3.29	5.3	9.13	14.7

Abbreviations: M, mean; SD, standard deviation; 95% CI, 95% confidence interval; ICC, intraclass correlation coefficient; SEM, standard error of measurement; SEM%, standard error of measurement as a percentage; MDC, minimum detectable change; MDC%, minimum detectable change as a percentage; N/A, not applicable; ^†^ Friedman test *p* values. Item-total correlation refers to the magnitude of association between each item and its domain. ** Significant correlation at *p* < 0.01. * Significant correlation at *p* ≤ 0.05.

## Data Availability

The datasets generated during and/or analyzed during the current study are available from the corresponding author upon reasonable request, the data are not publicly available due to privacy restrictions.

## Supplementary Materials

The following supporting information can be downloaded at: https://www.mdpi.com/article/10.3390/children13070906/s1, Supplementary Material S1: Children Self-Perceptions of Adequacy in and Predilection for Physical Activity (CSAPPA).

## Abbreviations

The following abbreviations are used in this manuscript:

95% CI	95% confidence interval
CFA	Confirmatory factor analysis
CFI	Comparative fit index
COSMIN	COnsensus-based Standards for the selection of health Measurement Instruments
CSAPPA	Children’s Self-Perceptions of Adequacy in and Predilection for Physical Activity
ICC	Intraclass correlation coefficient
MDC	Minimum detectable change
ML	Maximum likelihood
SD	Standard deviation
SEM	Standard error of measurement
SPLA-C	Spanish Physical Literacy Assessment for Children
PA	Physical activity
PE	Physical education
PL	Physical literacy
RMSEA	Root mean square error of approximation
TLI	Tucker–Lewis index
WLSMV	Weighted least squares mean and variance
