# Supplementary material for: Validity and Reliability of the Spanish Version of the Children’s Self-Perceptions of Adequacy in and Predilection for Physical Activity (CSAPPA) Questionnaire for Primary School Children Aged 6 to 12 Years
Source: Children (Basel). 2026 Jul 8;13(7):906. doi: 10.3390/children13070906 (PMC13406152; doi:10.3390/children13070906)
Supplement: Supplementary file 1 [file children-13-00906-s001.zip › children-4384347-supplementary.pdf]

Children Self-Perceptions of Adequacy in and Predilection for Physical Activity (CSAPPA)

Code:

What is most like you?	*That's not true for me*			*That's absolutely true for me*
1. When lessons finish, I can't wait to go out and play	1	2	3	4
2. I have a lot of fun in physical education lessons	1	2	3	4
3. I like games where you have to move around or run	1	2	3	4
4. I enjoy playing sport	1	2	3	4
5. I think physical education is the best subject of all	1	2	3	4
6. I'm good at games where you have to move around or run	1	2	3	4
7. I like sport	1	2	3	4
8. I always hurt myself whenever I do any sport	1	2	3	4
9. I like going out to play games where you have to move around or run	1	2	3	4
10. I'm good at most sports	1	2	3	4
11. I find it easy to learn games where you have to move around or run	1	2	3	4
12. I think I'm one of the best at sport	1	2	3	4
13. The games I play in physical education are difficult	1	2	3	4

14. I'd rather watch my teammates play	1	2	3	4
15. I'm one of the last to be picked to play	1	2	3	4
16. I like to take my time during break times	1	2	3	4
17. I enjoy my physical education lessons	1	2	3	4
18. I'm good enough at team sports	1	2	3	4
19. I like quiet games	1	2	3	4
20. I like going out at the weekends to play games that involve moving around or running	1	2	3	4
